# Deubiquitinase OTUD6A promotes proliferation of cancer cells via regulating Drp1 stability and mitochondrial fission

**DOI:** 10.1002/1878-0261.12825

**Published:** 2020-11-06

**Authors:** Le Shi, Jing Liu, Yunhua Peng, Jinfang Zhang, Xiangpeng Dai, Shuangxi Zhang, Yongyao Wang, Jiankang Liu, Jiangang Long

**Affiliations:** ^1^ Center for Mitochondrial Biology and Medicine The Key Laboratory of Biomedical Information Engineering of Ministry of Education School of Life Science and Technology and Frontier Institute of Science and Technology Xi'an Jiaotong University China; ^2^ Frontier Science Center for Immunology and Metabolism Medical Research Institute Wuhan University China; ^3^ Key Laboratory of Organ Regeneration and Transplantation of Ministry of Education First Hospital Jilin University Changchun China

**Keywords:** cancer cell growth, deubiquitination, Drp1, mitochondrial fission, OTUD6A

## Abstract

Dynamin‐related protein 1 (Drp1) is a cytosolic protein responsible for mitochondrial fission and is essential in the initiation and development of several human diseases, including cancer. However, the regulation of Drp1, especially of its ubiquitination, remains unclear. In this study, we report that the ovarian tumor‐associated protease deubiquitinase 6A (OTUD6A) deubiquitylates and stabilizes Drp1, thereby facilitating regulation of mitochondrial morphology and tumorigenesis. OTUD6A is upregulated in human patients with colorectal cancer. The depletion of *OTUD6A* leads to lower Drp1 levels and suppressed mitochondrial fission, and the affected cells are consequently less prone to tumorigenesis. Conversely, the overexpression of OTUD6A increases Drp1 levels and its protein half‐life and enhances cancer cell growth. Therefore, our results reveal a novel upstream protein of Drp1, and its role in tumorigenesis that is played, in part, through the activation of mitochondrial fission mediated by Drp1.

## Graphical abstract

OTUD6A deubiquitylates and stabilizes Drp1, thereby facilitating regulation of mitochondrial morphology and tumorigenesis. The depletion of *OTUD6A* leads to lower Drp1 levels and suppresses mitochondrial fission, and the affected cells are consequently less prone to tumorigenesis. Conversely, the overexpression of OTUD6A increases Drp1 levels and its protein half‐life and enhances cancer cell growth.

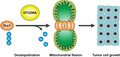

**Abbreviations**
Drp1: dynamin‐related protein 1
DUBs: deubiquitinases
His‐ub: His‐ubiquitin
IB: immunoblot
IHC: immunohistochemistry
IP: immunoprecipitation
MARCH5: membrane‐associated ring‐CH‐type finger 5
Mfn1: mitofusin 1
Mfn2: mitofusin 2
NIK: NF‐κB‐inducing kinase
Opa1: optic atrophy 1
OTUs: ovarian tumor‐associated proteases
OTUD6A: ovarian tumor‐associated protease deubiquitinase 6A

## 1 Introduction

Dynamin‐related protein 1 (Drp1), the ‘master regulator’ of fission, is involved in several important aspects of mitochondrial structure, and it functions by governing the processes of mitochondrial dynamics and providing necessary adenosine triphosphate to cells [1]. The advancements in molecular and cellular studies have revealed that changes in Drp1‐induced mitochondrial dynamics are involved in a number of biological processes, including cell division, embryonic development, autophagy apoptosis, metabolic processes, and cell death [2, 3]. Additionally, numerous studies have revealed that Drp1 overexpresses [4, 5, 6, 7] and is involved in the progress of various cancers [8, 9, 10].

The post‐translational modifications of Drp1 have been discovered to affect different aspects of Drp1 function, which is associated with cellular function [11, 12, 13]. Taguchi *et al*. [14] showed that Drp1 phosphorylation at Ser‐616 enhanced its activity, recruited Drp1 toward mitochondria, and initiated mitochondrial fission. Further study showed that Drp1 phosphorylation promoted cancer cell growth and suppressed tumorigenesis as well. Jung *et al*. [15] explained that NF‐κB‐inducing kinase (NIK) regulated Drp1 phosphorylation at Ser‐616 and dephosphorylation at Ser‐637, inducing mitochondrial fission, and promoting cell invasion. On the contrary, Li *et al*. [16] showed that the depletion of Yap droved Drp1 phosphorylation at Ser‐616 and dephosphorylation at Ser‐637, inducing mitochondrial fission and subsequently triggering cellular apoptosis and impaired cellular migration. The ubiquitination of Drp1 also regulates mitochondrial fission, which be further related to cell division. Membrane‐associated ring‐CH‐type finger 5 (MARCH5) is an E3 ubiquitin ligase that plays a major role in regulating Drp1 levels and mitochondrial division [14]. The ACP/Cdh1‐mediated ubiquitination of Drp1 regulates mitochondrial fragmentation at the end of mitosis [17]. Moreover, Parkin, an E3 ubiquitin ligase, has been shown to regulate the degradation of Drp1, leading to mitochondrial fission in the pathogenesis of Parkinson's disease [18].

It is well documented that the ubiquitination of many proteins can be reversed by deubiquitinases (DUBs), which play an important role in cell growth, apoptosis, and cancer [19, 20]. Approximately one hundred enzymes with deubiquitinating activity have been described, and a total of 16 ovarian tumor‐associated protease (OTU) members have been identified in human genome, making them the second largest DUB subfamily [20]. Numerous evidence has indicated that the majority of the OTU DUBs appear to have a crucial role in the origination and development of cancer. OTUB2 and OTUD2 have been identified displaying potential oncogenic traits in breast [21] and liver [22] cancer, respectively. On the other hand, some OTUs show at least potential tumor‐suppressive activity, including OTUD1 [23], OTUD3 [24], OTUD5 [25], and OTUD7A [26]. OTUD6A is a member of OTU classes. To date, OTUD6A has only been reported to be associated with p53 [27]. However, the physiology and biology activity of OTUD6A remain enigmatic.

Here, we investigate OTUD6A is a novel upstream of Drp1 and upregulated in patients with colorectal cancer, which suggested a potential mechanism linking OTUD6A to mitochondrial fission in cancer. These results uncover the importance of OTUD6A in tumorigenesis and new therapeutic strategies that attenuate mitochondrial fission through inhibition of the OTUD6A‐Drp1 pathway.

## 2 Materials and methods

### 2.1 Cell culture and plasmids

Multiple cell types, namely HEK293, HEK293T, HCT116, DLD1, and HeLa cells, were cultured in Dulbecco's modified Eagle medium (Life Technologies, Carlsbad, CA, USA) containing 10% fetal bovine serum (FBS), 100 units of penicillin, and 100 mg·mL^−1^ streptomycin. HeLa‐Drp1^−/−^ cells were constructed by CRISPR/Cas9 and kindly given by T. Zhang. All cell lines were previously measured for mycoplasma contamination.

Flag‐OTUB1, Flag‐OTUB2, Flag‐OTUD3, Flag‐OTUD5, Flag‐OTUD6A, Flag‐OTUD6B, and Flag‐OTUD7B constructs have been described previously [24]. The lentiviral vectors containing GFP and OTUD6A shRNA were generated with the guide sequences of 5′‐GCAAGCTGACCCTGAAGTTCAT‐3′ and 5′‐CATGATCTACTGCGACAACATC‐3′, respectively. A His‐ubiquitin (His‐ub) construct was as described previously [28]. HA‐Drp1 WT, K38A, S616A, MDVD truncation, Flag‐OTUD6A WT, and C152A were generated by cloning the corresponding cDNAs into the pcDNA3‐HA vector via BamHI and XhoI sites or pcDNA3‐Flag vector via XhoI and BamHI sites for this study.

### 2.2 Cell transfection and viral infection

For cell transfection, cells with 80% confluence were transfected using Lipofectamine (Invitrogen, Carlsbad, CA, USA) in Opti‐MEM medium (Invitrogen). Forty‐eight hours post‐transfection, the cells were harvested for analysis. For viral infection, the HEK293T cell line was used for packaging and amplifying the lentivirus. The media containing the viruses were collected at 48 and 72 h after transfection. Then, the media were filtered through a 0.45‐μμ filter, added with 4 μg·mL^−1^ polybrene (Sigma, St. Louis, MO, USA), and used for infecting the cells at 50% confluence. Forty‐eight hours postinfection, the cells were passaged and selected using 1 μg·mL^−1^ puromycin (Sigma) for 72 h to eliminate the uninfected cells before harvesting and subsequent western blotting analysis.

### 2.3 Antibodies and reagents

The antibodies used in this study are listed in Table 1. MG132 (BML‐PI102‐0005) was purchased from Enzo Life Sciences (Farmingdale, NY, USA). MitoTracker Red (M7513) was purchased from Invitrogen.

**Table 1:** Antibodies used in this study.

Antibody	Antibody source	Cat. no.	Specificity	Dilution
Drp1	Cell Signaling	8570	Ms	1 : 1000 1 : 200 (IF)
Mfn1	Cell Signaling	14739	Rb	1 : 1000
Mfn2	Cell Signaling	11925	Rb	1 : 1000
PTEN	Cell Signaling	9188	Rb	1 : 1000
Opa1	BD Biosciences	612607	Ms	1 : 1000
Drp1	Abcam	ab56788	Ms	1 : 200 (IHC)
OTUD6A	Abcam	ab185352	Rb	1 : 200 (IHC)
OTUD6A	ProteinTech	24486‐1‐AP	Rb	1 : 200 (IF)
OTUD6A	Novus Biological	NBP1‐91498	Rb	1 : 1000
HA	Santa Cruz	sc‐805	Rb	1 : 3000
HA	Covance	MMS‐101P	Ms	1 : 3000
Flag	Sigma	F7425	Rb	1 : 3000
Flag	Sigma	F3165	Ms	1 : 3000
Vinculin	Sigma	V9131	Ms	1 : 10 000

### 2.4 Immunoprecipitation or immunoblot

Cell lysates were collected according to a standard method described previously [29]. For immunoprecipitation (IP), 1 mg total lysates was incubated with the appropriate antibody‐conjugated beads (1–2 μg) for 4 h or overnight at 4 °C. For endogenous IP, the cell lysates were incubated with Drp1 antibody (1–2 μg) or IgG (2 μg) overnight at 4 °C, and protein A/G agarose beads (Pierce Biotechnology, Rockford, IL, USA) were then added and incubated for 1 h. The immunocomplexes were washed four times with NETN buffer (20 mm Tris, pH 8.0, 100 mm NaCl, 1 mm EDTA, and 0.5% NP‐40), subjected to SDS/PAGE, and further incubated with the indicated antibodies.

### 2.5 Immunohistochemistry and human tissue microarray

A total of 100 tissue samples, including 10 samples of normal tissues and 90 of colorectal cancer tissues, were purchased from Kexin Biotech Company (Co‐kx06c, Xi'an, China) and stained with human Drp1 or OTUD6A (dilution 1 : 200) for the immunohistochemistry (IHC) assay. Unfortunately, eight of colorectal cancer tissues were damaged during the IHC assay, the rest of 82 cancer tissues could be used for statistical analysis. The stained sections were scanned using a panoramic viewer (Budapest, Hungary). Detection and analysis of the staining were performed as described previously [29]. The stained cells were assessed according to the intensity (0 point: no; 1 point: weak; 2 points: moderate; and 3 points: strong) and the proportion of positive cells (0 point: 1–5%; 1 point: 6–25%; 2 points: 26–50%; 3 points: 51–75%; and 4 points: 76–100%). The assessment score was the point of intensity multiplied by the point for the proportion of positive cells, and the final score was the average of five views. The slides were examined independently by two pathologists blinded to the clinical and pathologic information. The project was authorized by the Ethics Committees of Xi'an Jiaotong University and conducted per the ethical standards of the Declaration of Helsinki.

### 2.6 Cell growth and colony formation assays

For cell growth, 1 × 10^4^ cells per well were plated in 6‐well plates. The cell number was calculated among 6 days. For colony formation, the cells were plated in 6‐well culture dishes (500 or 1 000 cells/well) and approved to growth for 10–14 days. The cells were stained with crystal violet, and the colony numbers were counted.

### 2.7 *In vitro* ubiquitination assay

Cells were transfected with His‐ub, Drp1, OTUD3, OTUD6A, OTUD6B, and OTUD7B. After transfection for 48 h, the cells were treated with MG132 (15 μm) overnight and then performed according to the previous description. The pull‐down proteins were subjected to SDS/PAGE for immunoblot (IB) analysis [29].

### 2.8 Fluorescence microscopy

The cells were seeded in a 12‐well plate with prepaved glass coverslips. For immunofluorescence, cells were incubated overnight at 4 °C with anti‐Drp1 and anti‐OTUD6A antibody and measured with Alexa Fluor 555‐labeled donkey anti‐mouse IgG (Beyotime, Shanghai, China) and FITC‐conjugated goat anti‐rabbit IgG (Invitrogen). Cells were subsequently stained with DAPI (Invitrogen) as a nuclear staining containing Antifade reagent (Bioworld Technology, St. Louis Park, MN, USA) followed by image acquisition using an inverted fluorescence microscope (Olympus IX71, Tokyo, Japan). The colocalization was analyzed by the imagej software (NIH, Bethesda, MD, USA). Manders' colocalization coefficients were chosen for the colocalization ratio. Manders' M1 (M2) represents the proportion of red (green) fluorescence overlapped with green (red) fluorescence in total red (green) fluorescence. For fluorescence microscopy of mitochondria, cells with 80% confluence were incubated with MitoTracker Red (100 nm) for 30 min at 37 °C and then washed with PBS followed by image acquisition using an inverted fluorescence microscope (Olympus IX71). The mean mitochondrial length was determined by measuring mitochondrial from cells obtained by fluorescence microscopy using the imagej software (NIH). Fifteen cells from three independent experiments were analyzed for quantification.

### 2.9 Mouse xenograft assays

BALBc_nu mice were injected with 5 × 10^6^ OTUD6A^−/−^ HCT116 cells as previously described [30]. Tumor size was tested every 4 days with a vernier caliper, and the tumor volume was calculated with the formula: *L* × *W*2 × 0.52 (*L*, the longest diameter; *W*, the shortest diameter). After 20 days, mice were sacrificed, xenografted solid tumors were dissected, and then tumor weights were measured. BALBc_nu female nude mice (4–5 weeks of age) were purchased from the SLAC Laboratory Animal Co., Ltd (Shanghai, China), following the principles of the National Institutes of Health on the use of laboratory animals, with a protocol that was approved by the University of Xi'an Jiaotong Institutional Animal Care and Use Committee.

### 2.10 Statistics

The results are presented as the mean ± SD. graphpad software (GraphPad Software Inc., La Jolla, CA, USA) was used to analyze data. Unpaired, two‐tailed Student's *t*‐test was used to evaluate data from two groups. One‐way or two‐way ANOVA was used to assess other data, followed by a Bonferroni *post hoc* test. *P* < 0.05 was considered to indicate statistical significance.

## 3 Results

### 3.1 OTUD6A interacts with Drp1

**Fig. 1:**
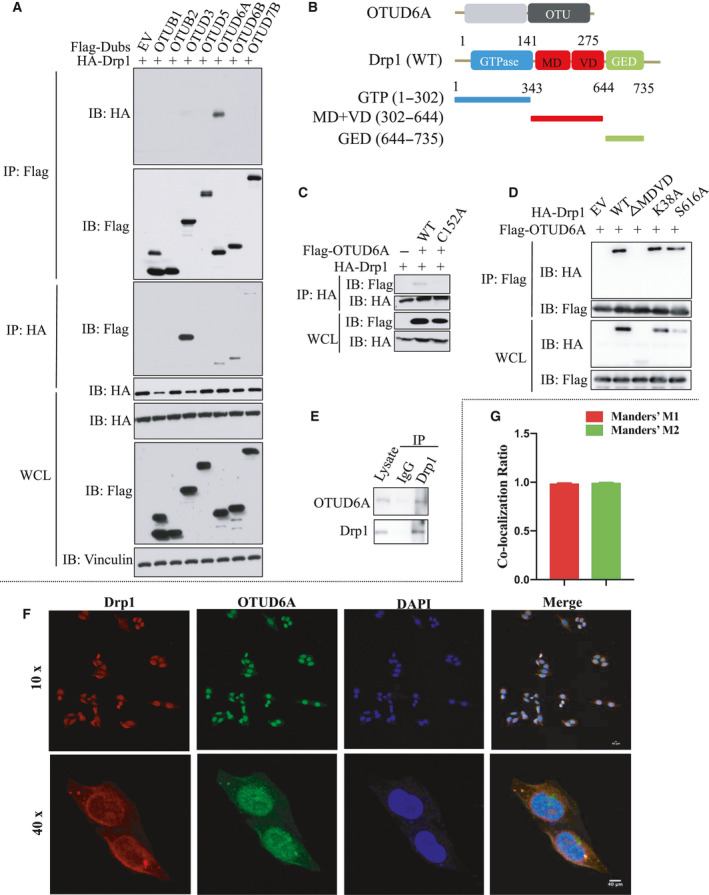
OTUD6A interacts with Drp1. (A) Drp1 specifically interacts with OTUD6A in cells. IB analysis of IP and WCL derived from HEK293 cells transfected with HA‐Drp1 and the indicated Flag‐DUBs for 48 h. (B) Overview of OTUD6A and Drp1 structures. (C) OTUD6A WT but not mutant C152A specifically interacts with Drp1. (D) HEK293 cells transfected with the Drp1 constructs were subjected to IP with the anti‐HA or anti‐Flag antibodies. (E) Drp1 interacts with endogenous OTUD6A in cells. HEK293 cell lysates were subjected to pull down by anti‐Drp1 antibody and protein A/G agarose, followed by IB analysis with the indicated antibodies. (F) HeLa cells were fixed and immunostained with anti‐Drp1 and anti‐OTUD6A before confocal microscopy. The magnification of the images is 400×. (G) Quantification of the colocalization between Drp1 and OTUD6A. Scale bar represents 40 μm.

We firstly identified the DUBs that could be responsible for deubiquitinating Drp1 by screening a panel of OTU scaffolding proteins to identify the potential complex for Drp1. Notably, OTUD6A intensively interacted with Drp1 *in vitro* using Flag IP, while OTUD3, OTUD6A, OTUD6B, and OTUD7B interacted with Drp1 *in vitro* using HA IP. However, OTUD6A both interacted with Drp1 using the two agarose beads (Fig. 1A). OTUD6A contained only one domain, the OTU domain, which is important for its interaction with most of its substrate proteins (Fig. 1B). C152 is an active site of OTUD6A (www.uniprot.org/uniprot/Q7L8S5). We constructed the mutant C152A OTUD6A and found that the mutant C152A lost its ability to interact with Drp1 (Fig. 1C). This indicated that the mutant C152A might be a catalytically inactive mutant of OTUD6A. K38 is an dominant and inactivated mutation site, which promotes fusion [31]. S616 is a phosphorylation site, which initiates mitochondrial fission. Drp1 contained three domains, including a highly conserved N‐terminal GTPase domain, a helical MDVD domain in the middle, and a GED domain at the C‐terminal (Fig. 1B). We found that the K38A and S616A mutants of Drp1 did not impact the binding of OTUD6A, MDVD domain of Drp1 to be the critical interacting region of OTUD6A protein (Fig. 1D). Next, we investigated whether endogenous Drp1 interacted with endogenous OTUD6A. Importantly, we found that endogenous OTUD6A and Drp1 proteins co‐immunoprecipitated (Fig. 1E). We further detected the interaction between Drp1 and OTUD6A by a confocal microscopy analysis, and results showed that the two proteins were mostly colocalized, and the colocalization ratio was 0.987 (Manders' M1) and 0.994 (Manders' M2), respectively (Fig. 1F,G).

### 3.2 OTUD6A deubiquitinates and stabilizes Drp1

**Fig. 2:**
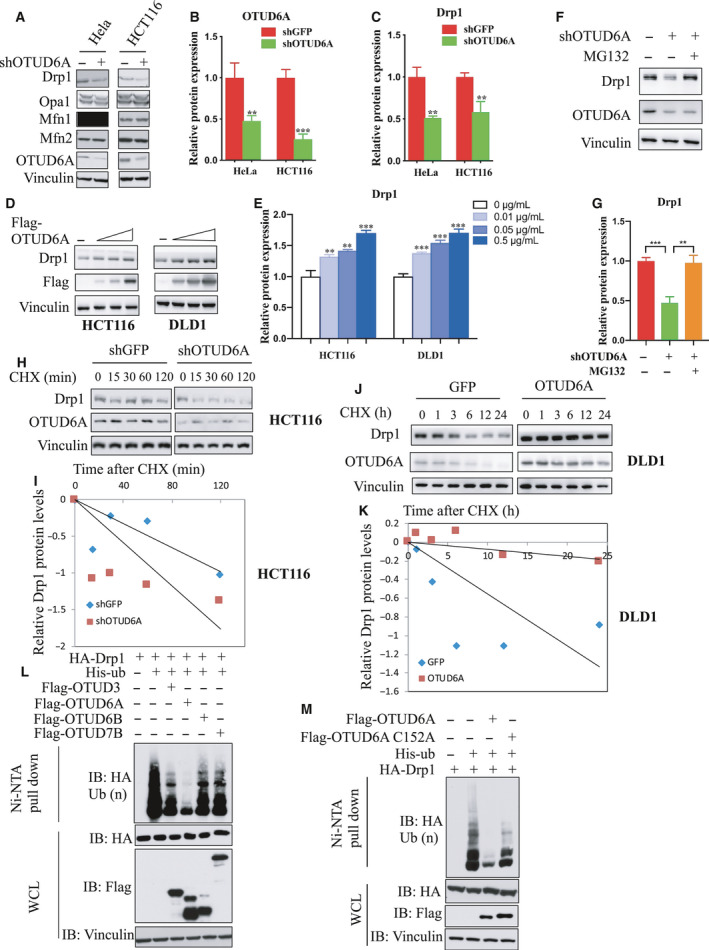
OTUD6A deubiquitinates and affects Drp1 stability. Cells were infected with pLKO‐shOTUD6A or mock virus, selected by puromycin for 3 days to eliminate noninfected cells. (A) Depletion of OTUD6A in cells leads to decreased Drp1. Relative OTUD6A (B) and Drp1 (C) expression of (A) was further quantified. (D) Exogenous expression of OTUD6A causes the accumulation of Drp1 in HCT116 (left) and DLD1 (right) cells. (E) Relative Drp1 expression of (D) was further quantified. (F) HCT116 cells transfected with pLKO‐shOTUD6A were left untreated or treated with proteasome inhibitor MG132 (10 μm, 12 h), and the proteins were extracted and subjected to western blotting. (G) Relative Drp1 expression of (F) was further quantified. (H, I) Half‐life analysis of Drp1 in HCT116 cells with and without knockdown of OTUD6A. (J, K) Half‐life analysis of Drp1 in DLD1 cells expressing OTUD6A. (L) OTUD6A inhibits Drp1 ubiquitination in cells. IB analysis of Ni‐NTA pull‐down products and WCL derived from HEK293 cells transfected with the indicated constructs. (M) OTUD6A WT but not the mutant C152A deubiquitinates Drp1. The mean ± SD for three independent experiments is shown. For two groups, data were analyzed by unpaired, two‐tailed Student's *t*‐test. **P < 0.01, ***P < 0.001. Other data were analyzed by one‐way ANOVA, followed by a Bonferroni *post hoc* test. **P < 0.01, ***P < 0.001.

To test the effect of OTUD6A on Drp1 stability, we measured the expression of Drp1 in cells infected with OTUD6A shRNA by western blotting. The depletion of *OTUD6A* markedly decreased Drp1 levels, whereas the levels of optic atrophy 1 (Opa1), mitofusin 1 (Mfn1), and mitofusin 2 (Mfn2), the other key regulators of mitochondrial dynamics, were not affected (Fig. 2A–C). On the other hand, the overexpression of OTUD6A in HCT116 and DLD1 cells resulted in Drp1 elevation (Fig. 2D,E). This indicated that OTUD6A likely affects Drp1 protein turnover. To identify whether proteasomal responsible for OTUD6A‐mediated Drp1 degradation, the proteasomal inhibitor MG132 was used to inhibit the pathway of protein degradation. As shown in Fig. 2F,G, the decrease in Drp1 was reversed by the addition of the MG132. We also performed a protein half‐life assay to examine the effect of OTUD6A on Drp1 stability with the protein synthesis inhibitor cycloheximide (CHX). The half‐life of Drp1 was shortened in cells depleted of *OTUD6A* (Fig. 2H,I and Fig. S1A,B). Additionally, the half‐life of Drp1 was prolonged in cells overexpressing OTUD6A (Fig. 2J,K and Fig. S1C,D). These results demonstrate that OTUD6A regulates Drp1 stability.

To investigate whether OTUD6A could remove the ubiquitin chain of Drp1, the OTUD subclasses were determined by ubiquitination assays in vitro. The result showed that OTUD6A could obviously deubiquitinated Drp1 (Fig. 2L). Interestingly, OTUD3 also interacts with Drp1 (Fig. 1A) and slightly decrease Drp1 ubiquitination (Fig. 2L). However, Drp1 levels were not altered in the cells with OTUD3 shRNA. As PTEN is a proved substrate of OTUD3 [24], we detect the protein level of PTEN for identifying the cells with knockdown of OTUD3 (Fig. S1E). Next, we used OTUD6A catalytically inactive (C152A) mutant. The ubiquitylation assay showed that WT but not C152A mutant OTUD6A obviously deubiquitinated Drp1 (Fig. 2M).

### 3.3 Drp1 and OTUD6A are aberrantly upregulated in colorectal cancer tissues

**Fig. 3:**
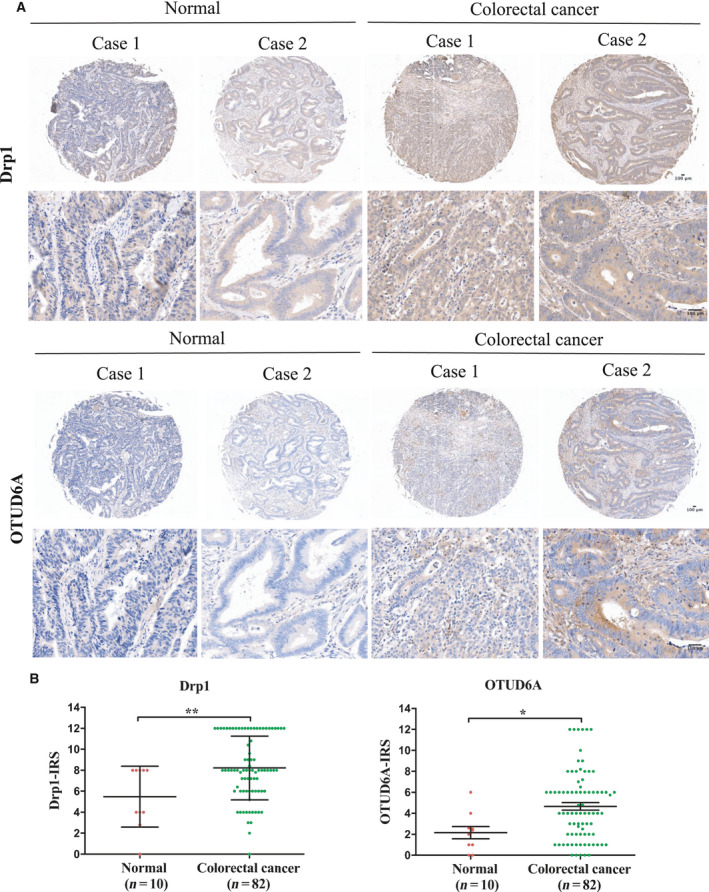
Drp1 and OTUD6A exhibit higher expression in colon cancer tissues than normal tissues. (A) Representative images from immunohistochemical staining of Drp1 (upper) and OTUD6A (lower) in colon cancer tissues. The magnification of the upper and lower images is 50× and 200×, respectively. (B) Staining of (A) was assessed, calculated, plotted, and analyzed. Data were analyzed by unpaired, two‐tailed Student's *t*‐test. *P < 0.05, **P < 0.01. Scale bar represents 100 μm.

The previous studies showed that an increase in the fission protein Drp1 was found in breast, lung, and oncocytic thyroid cancers compared with matched nonmalignant tissues [5, 6, 7]. Our results found that the expression of Drp1 was increased in human colorectal cancer (Fig. 3). The above results revealed that OTUD6A could regulate Drp1 stability, while the physiological role of OTUD6A in tumors has not yet been extensively investigated. To uncover the potential function of OTUD6A in cancer, we firstly performed IHC analysis to assess the OTUD6A protein levels in cancer tissues and found that OTUD6A was aberrantly upregulated in colorectal cancer tissues compared with normal tissues (Fig. 3). The demonstration of OTUD6A and Drp1 expression in colorectal tumor and the interaction between the two proteins support our hypothesis that OTUD6A might play a promotive role in cancer progression.

### 3.4 OTUD6A affects cell growth

**Fig. 4:**
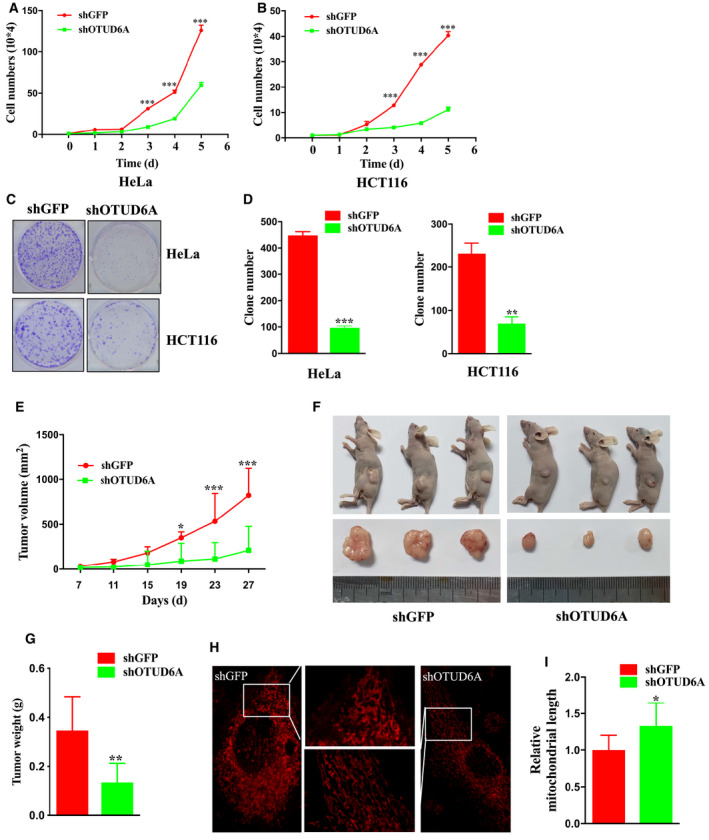
OTUD6A affects cell growth. Cells were infected with pLKO‐shOTUD6A or mock virus, selected by puromycin for 3 days to eliminate noninfected cells. (A) Growth curve of indicated cells. (B) Colony formation was carried out with the indicated cells. (C) Relative colony numbers were further quantified for colony formation. (D–F) Stable HCT116 cell lines with/without knockdown of OTUD6A were used for xenograft assays, and tumor volume (D) was monitored every four days. The tumors were dissected (E) and were weighed in (F) (*n* = 6). (G) Fluorescence staining of mitochondrial morphology in HeLa cells with/without knockdown of OTUD6A. The magnification of the images is 400×. (H) The length of mitochondrial fragments in cells treated with shOTUD6A relative to cells treated with shGFP. The mean ± SD for three independent experiments is shown. Data were analyzed by unpaired, two‐tailed Student's *t*‐test. *P < 0.05, **P < 0.01, ***P < 0.001. Scale bar represents 40 μm.

To assess the biological roles of OTUD6A in cancer, we inhibited the expression of OTUD6A in cells with shRNA (Fig. 2A). We observed that the deletion of *OTUD6A* could dramatically attenuate cell proliferation (Fig. 4A) and colony formation (Fig. 4B, C) in vitro and xenograft growth *in vivo* (Fig. 4D–F). Conversely, stably expressed OTUD6A (Fig. S2A) could apparently enhance cancer cell biological behaviors, including proliferation (Fig. S2B) and colony formation (Fig. S2C,D). Overexpression of the C152A mutant of OTUD6A could also impact cell growth but it is slight and not stronger than OTUD6A (Fig. S2A–D), suggesting that other signaling pathways independent of Drp1 might be involved in OTUD6A‐mediated cancer cell growth.

Mitochondrial dynamics are related to the initiation and development of cancer, and Drp1 has a critical role in mitochondrial morphology. As OTUD6A regulates Drp1 stability which has been revealed above, we next suppose that OTUD6A could affect mitochondrial dynamics. To investigate whether OTUD6A affects mitochondrial dynamics, we examined mitochondrial morphology in cells knocked down with OTUD6A. Fluorescence microscopy revealed that the depletion of OTUD6A resulted in less fragmented mitochondria. The length of the mitochondria was also significantly increased in HeLa cells transfected with OTUD6A shRNA (Fig. 4G, H). These results indicate that dysregulation of OTUD6A can alter mitochondrial morphology.

### 3.5 Cancer suppression induced by OTUD6A can be partly restored by Drp1 expression

**Fig. 5:**
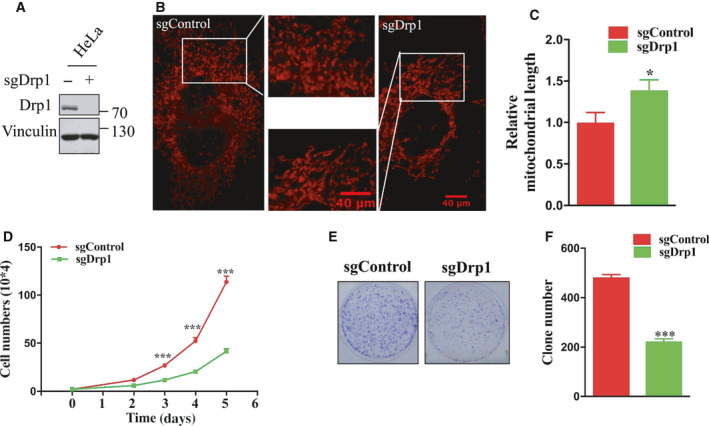
Drp1 affects cell growth. (A) HeLa cells deleted of *Drp1* were achieved by CRISPR/Cas9 and detected by western blotting. (B) Fluorescence staining of mitochondrial morphology in cells with/without knockout of Drp1. The magnification of the images is 400×. (C) The length of mitochondrial fragments in cells treated with sgDrp1 relative to cells treated with sgControl. (D) Cell growth of indicated cells. (E) Colony formation of the indicated cells. (F) Relative colony numbers were further quantified to determine the extent of the colony formation. The mean ± SD for three independent experiments is shown. Data were analyzed by unpaired, two‐tailed Student's *t*‐test. *P < 0.05, ***P < 0.001. Scale bar represents 40 μm.

**Fig. 6:**
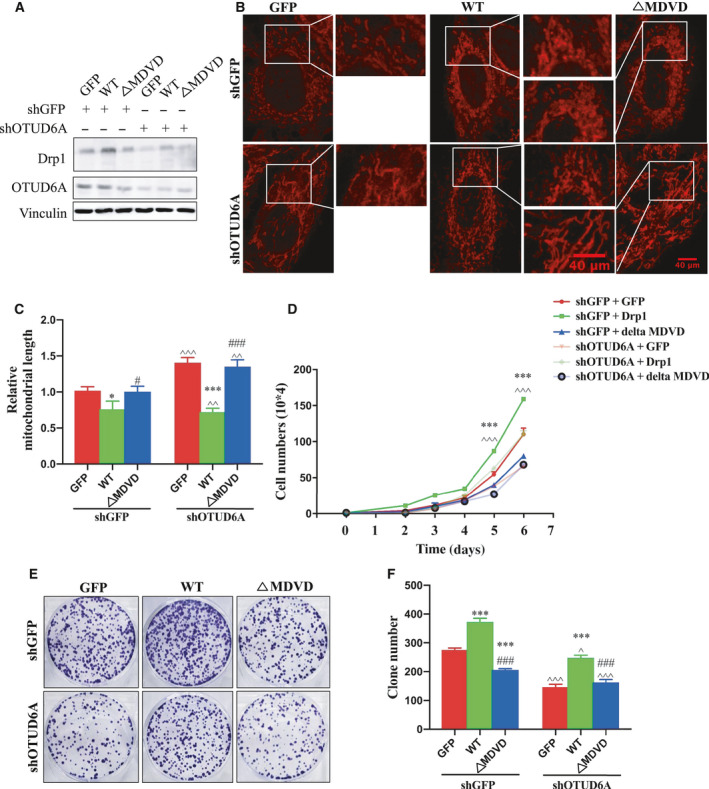
Decreased cancer cell growth induced by OTUD6A can be partly restored by Drp1 expression. (A) HeLa cells were infected with pLKO‐shOTUD6A, selected by puromycin for 3 days. OTUD6A^−/−^ cells were then infected with Drp1‐WT or Drp1‐ΔMDVD, selected by hygro for 3 days. (B) Fluorescence staining of mitochondrial morphology in HeLa cells stably knockdown OTUD6A and express Drp1‐WT or Drp1‐ΔMDVD. The magnification of the images is 400×. (C) The length of mitochondrial fragments in the indicated HeLa cells. (D) Cell growth of indicated cells. (E) Colony formation of the indicated cells. (F) Relative colony numbers were further quantified to determine the extent of the colony formation. The mean ± SD for three independent experiments is shown. Data were analyzed by one‐way or two‐way ANOVA, followed by a Bonferroni *post hoc* test. *P < 0.05, **P < 0.01, ***P < 0.001. For image C and F, *P < 0.05, ***P < 0.001 GFP vs WT. ^#^ *P* < 0.05, ^###^ *P* < 0.001 WT vs. ΔMDVD; ^^^ *P* < 0.05, ^^^^ *P* < 0.01, ^^^^^ *P* < 0.001 shGFP + GFP vs. shOTUD6A. For image D, ***P < 0.001 shGFP + GFP vs. shOTUD6A + GFP; ^^^^^ *P* < 0.001 shGFP + GFP vs. shOTUD6A + ΔMDVD. Scale bar represents 40 μm.

Given that the Drp1 protein is changed in OTUD6A‐mediated tumors, we next examined whether Drp1 was involved in the molecular signaling pathway. It has been revealed that Drp1, a mitochondrial fission protein, plays a crucial role in cancer cell growth, migration, and invasion [10, 32]. Consistent with previous findings, we observed that the deletion of *Drp1* could markedly attenuate mitochondrial fission, cell proliferation, and colony formation (Fig. 5A–F). Aligned with this demonstration, the overexpression of Drp1 caused an increase in cancer cell biological behaviors (Fig. S3). These findings support the notion that Drp1 plays an oncogenic role in tumorigenesis. Notably, we found that the OTUD6A shRNA‐induced lower drp1 levels and fragmented mitochondria could be blocked by exogenous expression of Drp1 in HeLa cells (Fig. 6A–C). More importantly, OTUD6A shRNA‐induced cell growth reduction could also be reversed by overexpression of Drp1 (Fig. 6D–F). However, the exogenous expression of the Drp1‐ΔMDVD did not restore the suppression of cancers induced by OTUD6A (Fig. 6A–F). Altogether, these results suggest that OTUD6A‐induced cancer cell growth was partly Drp1‐dependent and Drp1 MDVD domain is critical in this signaling pathway.

## 4 Discussion

**Fig. 7:**
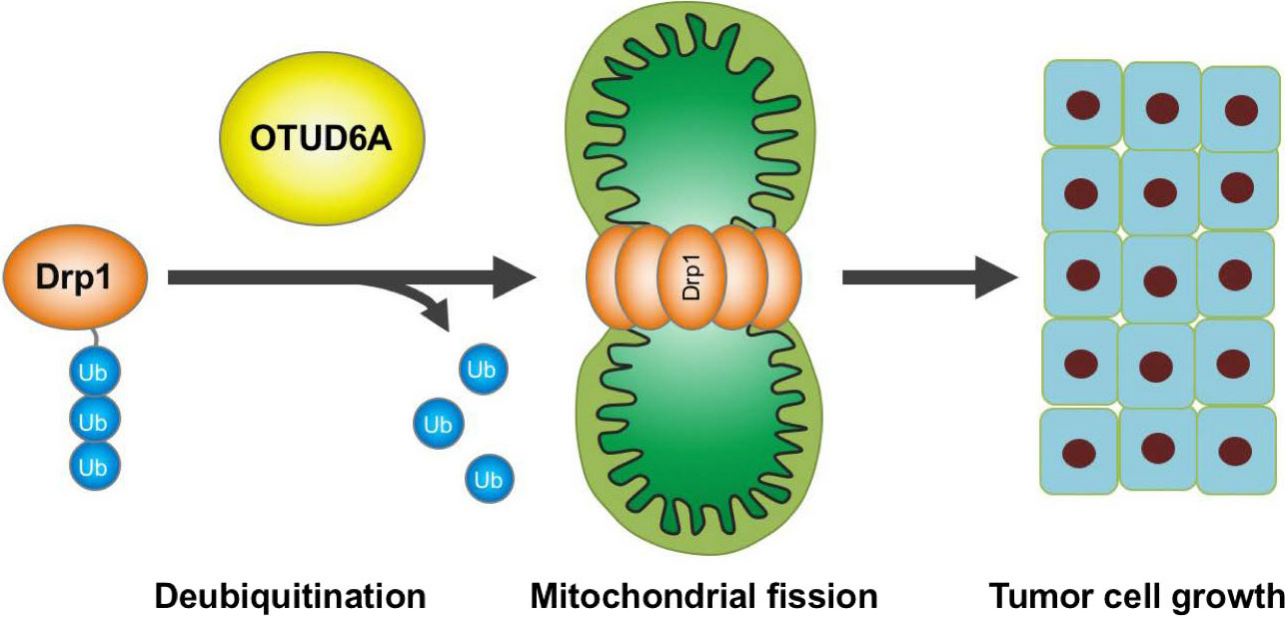
A schematic diagram of the possible mechanism of OTUD6A‐induced promotion of cell growth. OTUD6A might interact with Drp1, regulate the stability of Drp1 and subsequent mitochondrial fission, and eventually accelerate tumor cell growth.

The current study reveals that OTUD6A might play an oncogenic role in tumorigenesis and that OTUD6A regulates Drp1 stability and mitochondrial fission. Our results implicate a potential role for OTUD6A dysregulation in mitochondrial dynamics and in tumor cell growth (Fig. 7).

Numerous studies have revealed a connection between ubiquitin and protein [33, 34]. Similar to the balance of phosphorylation events by phosphatases, ubiquitination is counteracted by deubiquitinases. Many DUBs are associated with tumors by altering their protein expression. For instance, increased expression levels of OTUD6B, UCH37, VCPIP1, USP7, and COPS5 have been detected in various breast cancers [35]. Furthermore, a number of OTU DUBs that regulate important cell signaling pathways have been identified, and have been associated with various human diseases [20, 36, 37], whereas the physiological and the biology events of OTUD6A remain unknown. Here, we show a straightforward mechanism for how OTUD6A impacts mitochondrial morphology, leading to tumor cell growth. Strong evidence has shown that the accumulation of mitochondrial fission leads to mitochondrial fragments, increases neuronal apoptosis, and associates with neurodegenerative diseases [13]. While numerous studies also point out mitochondrial fission involved in tumorigenesis [12], these discrepancies indicate that mitochondrial fission plays different roles depending on the various conditions.

Drp1‐mediated mitochondrial fission is essential for the completion of cytokinesis and for the proper distribution of mitochondria into daughter cells [38, 39]. Furthermore, Drp1 protein is highly expressed in breast, lung, and oncocytic thyroid tumors, and promotes the progress of various cancers, which indicates that Drp1 can function as a tumor oncogene. Surprisingly, OTUD6A deubiquitinated and stabilized Drp1. We speculate that OTUD6A is one of the factors that mediate tumorigenesis. Based on the results presented above, OTUD6A might function as a tumor oncogene through its action toward mitochondrial fission. In addition, studies indicated that Drp1 plays important role in the cell cycle [40], cisplatin resistance [41], and glucose metabolism [42] of ovarian cancer, which were all associated with ovarian cancer cell growth. Moreover, OTUD6A is a member of OTU families. Therefore, according to the results presented here, it is possible that OTUD6A also has an oncogenic role in ovarian cancer via regulating Drp1. However, Kim *et al*. discovered that Drp1 was dramatically reduced in colon and lung cancer tissues, whereas no change in Drp1 was observed in breast or prostate tumors, a finding contrary to other findings [6, 7]. In fact, they pointed out that Drp1 levels in colon cancers were different in males and females. A total of 87.5% of male patients were observed to have low levels of Drp1, whereas half of the colon cancer tissues in female patients had decreased Drp1 levels. Furthermore, they did not show the age of the tissues used in their experiments, and it is possible that Drp1 levels varied with patient age. In addition, there were only 12 colon cancer tissues used in their study, and only three samples of grade III showed significantly reduced Drp1 levels [43].

The post‐translational modifications of Drp1 regulate its activity and mitochondrial function. Gu *et al*. [44] have reported that glyceronephosphate O‐acyltransferase recruits deubiquitinase USP30 and further stabilizes Drp1 to promote hepatocarcinogenesis. In support of this, our study has uncovered a novel regulatory pathway of Drp1 by deubiquitination and stability involving OTUD6A. In addition, USP9X is known to regulate the invasion of prostate cancer cells by inducing ERK‐mediated Drp1 phosphorylation, but not the stability [45], suggesting that deubiquitinase may affect the phosphorylation of Drp1 and mitochondrial dynamics. However, in our study, the Drp1‐S616A mutant had no effect on the binding of OTUD6A (Fig. 1D) and cell proliferation (data not shown). To some extent, this study explains our results that the suppression induced by OTUD6A could be mildly reversed by the overexpression of Drp1, which indicates other posttranslational modifications of Drp1 and mechanism might be involved. Interestingly, MDVD domain, a helical domain, has been demonstrated to mutate easily [46]. A recently reported mutation in Drp1 resulted in lethality marked by microcephaly and metabolic aberrations [47], which indicated that the MDVD domain of Drp1 might play a crucial role in cancer processes. We found that missing MDVD domain truly affects the binding of OTUD6A and the cancer cell proliferation.

Recent combinatorial studies have indicated the great potential of DUBs as novel targets in strategies to combat cancer. We provide evidence that the deletion of *OTUD6A* reduces mitochondrial fragmentation, inhibits tumor cancer cell proliferation, and impairs xenograft growth. Accordingly, small molecule inhibitors against OTUD6A might be potential drugs in the field of anticancer therapies. Interestingly, the diterpenoid derivative 15‐oxospiramilactone showed that it could inhibit DUB USP30, resulting in the elevated Mfn protein levels that promote mitochondrial fusion [48]. However, there is no inhibitor reported for OTU DUBs, and it still remains to be explored.

## 5 Conclusions

Mitochondrial dynamics have an crucial effect on the abnormal of mitochondrial function and can increase cell oxidative stress. As the key signaling protein regulating mitochondrial fission, Drp1 has been found to be tightly related to various cancers. To explore, the regulation of Drp1 signaling pathway will help understand the initiation and progression of cancer and improve the development of new targeted drug. Our study demonstrates a novel OTUD6A/Drp1 axis, and its role in tumorigenesis, partly via regulating Drp1 stability and mitochondrial fission. However, future studies are needed to deeply explore the mechanism of OTUD6A regulating Drp1 and determine the physiological functions of OTUD6A in tumorigenesis, for example, those using OTUD6A‐knockout mice.

## Conflict of interest

The authors declare no conflict of interest.

## Author contributions

LS, JGL, and JKL designed the study; LS performed the experiments with the help of JL, YHP, and SXZ, YW; LS, and JGL analyzed the data and wrote the manuscript; and LS, JGL, JKL, JFZ, and XPD revised the manuscript.

## Supporting information


**Fig. S1.** The stability of Drp1.

**Fig. S2.** Overexpression of OTUD6A affects cell growth.

**Fig. S3.** Overexpression of Drp1 affects cell growth.

Supplementary Material
