# Supplementary figures and images for: Deubiquitinase OTUD6A promotes proliferation of cancer cells via regulating Drp1 stability and mitochondrial fission
Source: Mol Oncol. 2020 Nov 6;14(12):3169–83. doi: 10.1002/1878-0261.12825 (PMC7718948; doi:10.1002/1878-0261.12825)

**A**

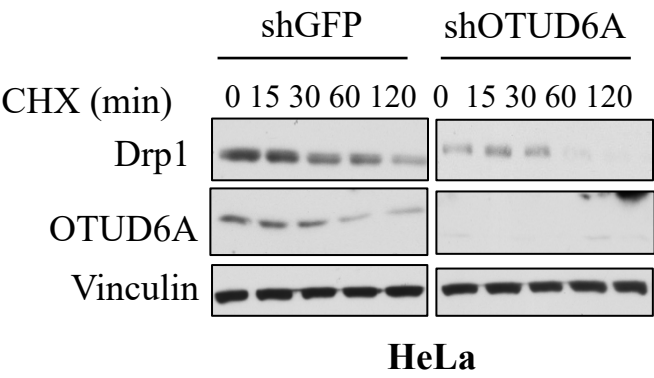

**B**

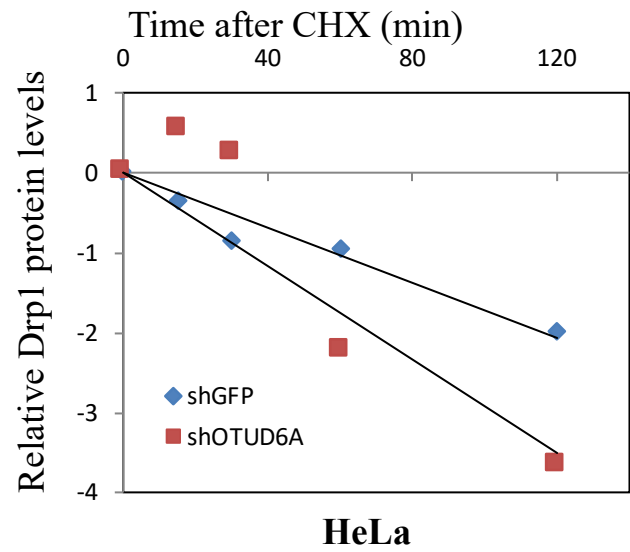

**E**

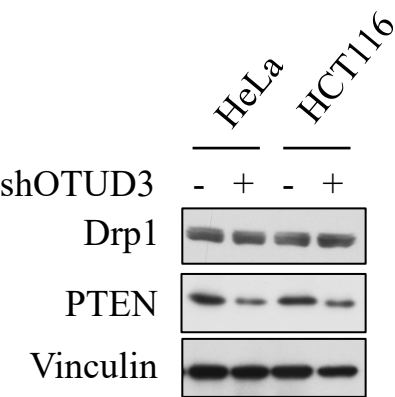

**C**

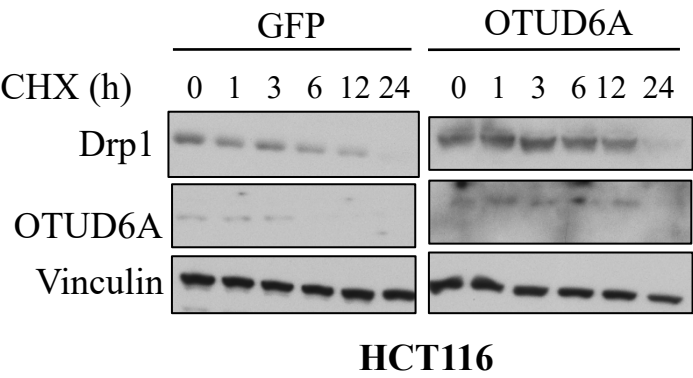

**D**

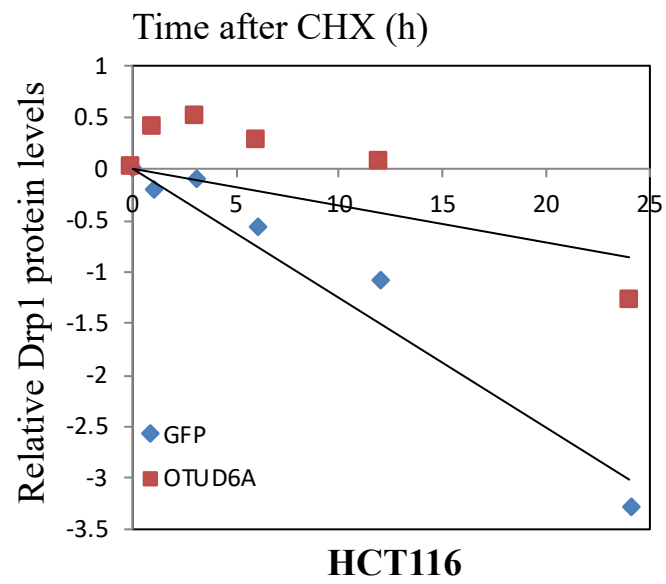

Supplement: Supplementary file 1 — Fig. S1. The stability of Drp1. [file MOL2-14-3169-s001.pdf]

A

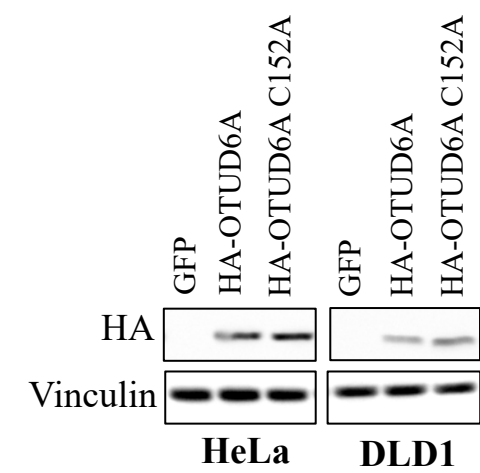

B

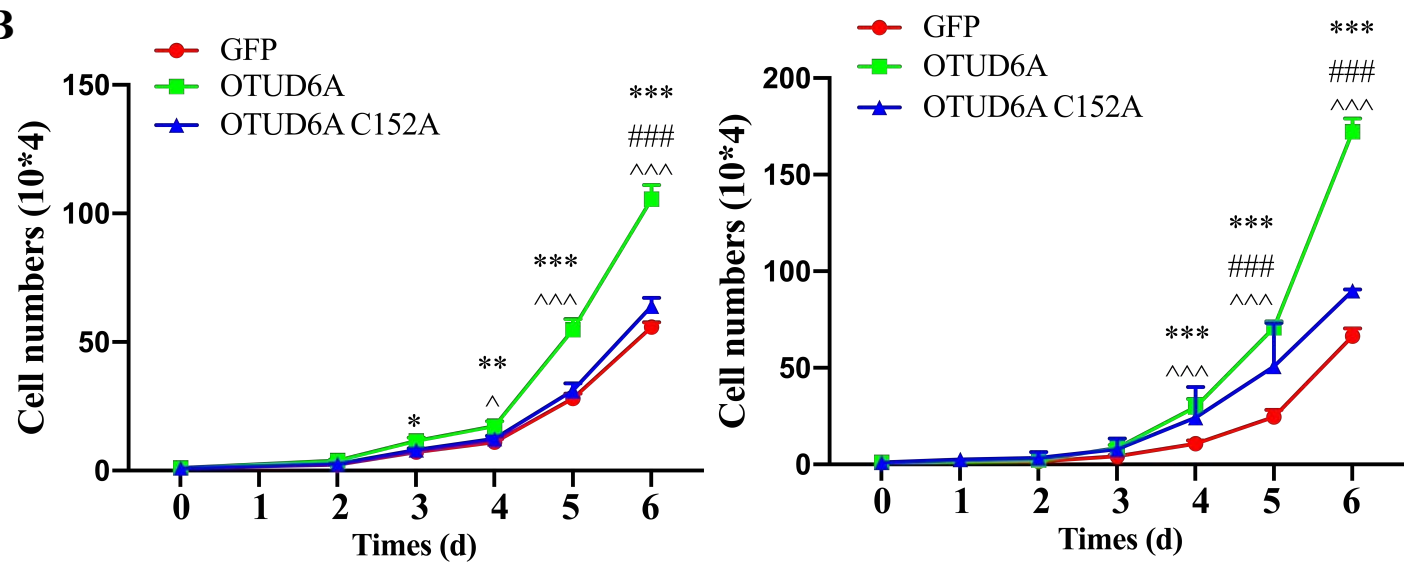

C

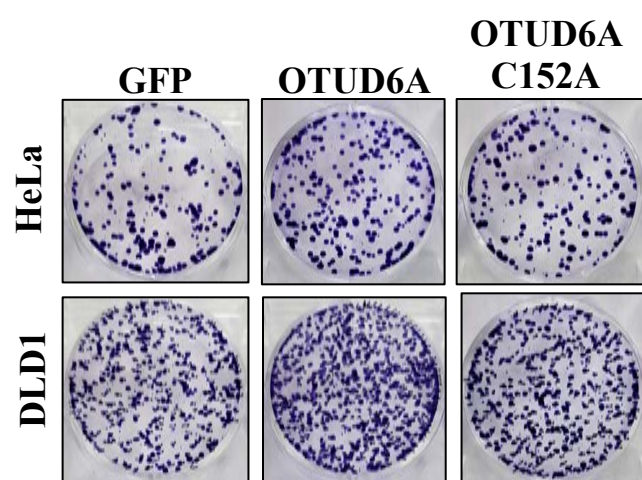

D

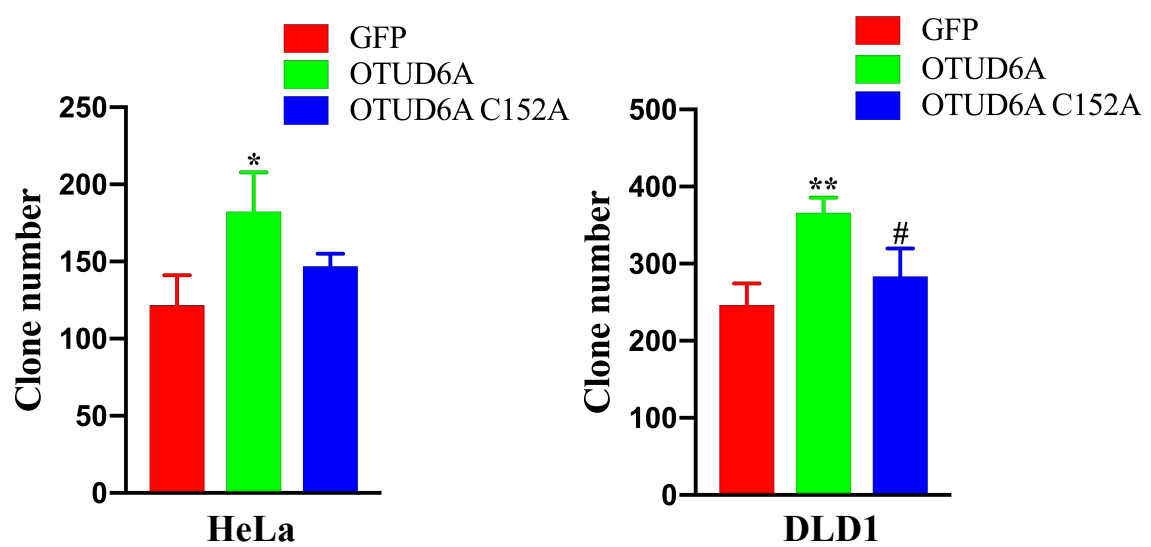

Supplement: Supplementary file 2 — Fig. S2. Overexpression of OTUD6A affects cell growth. [file MOL2-14-3169-s002.pdf]

**A**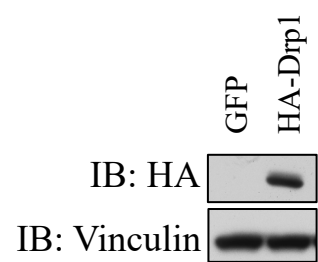**B**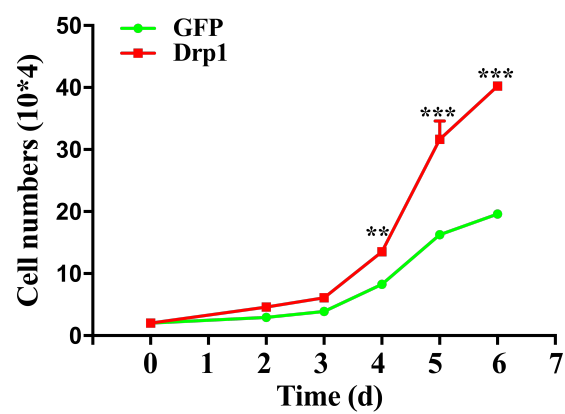**C**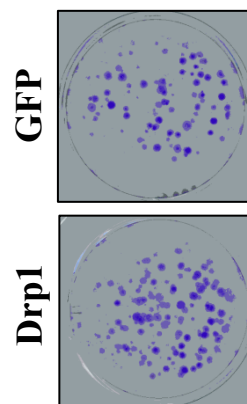**D**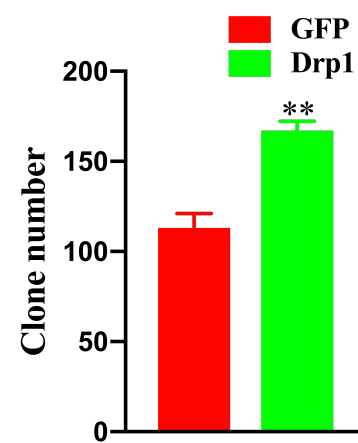

Supplement: Supplementary file 3 — Fig. S3. Overexpression of Drp1 affects cell growth. [file MOL2-14-3169-s003.pdf]
