# Supplementary material for: Deubiquitinase OTUD6A promotes proliferation of cancer cells via regulating Drp1 stability and mitochondrial fission
Source: Mol Oncol. 2020 Nov 6;14(12):3169–83. doi: 10.1002/1878-0261.12825 (PMC7718948; doi:10.1002/1878-0261.12825)
Supplement: Supplementary file 4 — Supplementary Material [file MOL2-14-3169-s004.docx]

**Fig. S1.** **The stability of Drp1.** (A, B) Half-life analysis of Drp1 in HeLa cells with and without knockdown of OTUD6A. (C, D) Half-life analysis of Drp1 in HCT116 cells expressing OTUD6A. (E) HeLa and HCT116 cells were infected with pLKO-shOTUD3 or mock virus, selected by puromycin for 3 days to eliminate non-infected cells, and subjected to IB analysis with the indicated antibodies.

**Fig. S2. Overexpression of OTUD6A affects cell growth.** (A-D) HeLa and DLD1 cells were infected with OTUD6A or OTUD6A C152A, selected by hygro for 3 days to eliminate non-infected cells, and subjected to IB analysis with the indicated antibodies. (A) HeLa and DLD1 cells stably express OTUD6A. (B) Growth curve of indicated cells. (C) Colony formation was carried out with the indicated cells. (D) Relative colony numbers were further quantified for colony formation. The mean ± SD for three independent experiments are shown. Data were analysed by one-way ANOVA, followed by a Bonferroni *post hoc test*. For image B, **P* < 0.05, ***P* < 0.01, ****P* < 0.001 OTUD6A vs. GFP; ^###^*P* < 0.001 OTUD6A C152A vs. GFP; ^*P* < 0.05, ^^^*P* < 0.001 OTUD6A C152A vs. OTUD6A. For image D, **P* < 0.05, ***P* < 0.01 OTUD6A vs. GFP; ^#^*P* < 0.05 OTUD6A C152A vs. OTUD6A.

**Fig. S3. Overexpression of Drp1 affects cell growth.** (A-D) HeLa cells were infected with Drp1, selected by hygro for 3 days to eliminate non-infected cells, and subjected to IB analysis with the indicated antibodies. (A) HeLa cells stably express Drp1. (B) Growth curve of indicated cells. (C) Colony formation was carried out with the indicated cells. (D) Relative colony numbers were further quantified for colony formation. The mean ± SD for three independent experiments are shown. Data were analysed by unpaired, two-tailed Student’s *t*-test. ***P* < 0.01, ****P* < 0.001.
